# Analysis of proteins by a radical-free and highly reducing method of two-dimensional polyacrylamide gel electrophoresis

**DOI:** 10.3389/fmolb.2026.1777271

**Published:** 2026-04-24

**Authors:** Akira Wada, Masami Ueta, Chieko Wada, Yasushi Maki, Takehito Tanzawa, Hideji Yoshida

**Affiliations:** 1 Biological Information Research, Yoshida Biological Laboratory Inc., Kyoto, Japan; 2 Department of Physics, Osaka Medical and Pharmaceutical University, Osaka, Japan; 3 Institute for Protein Research, The University of Osaka, Osaka, Japan

**Keywords:** 100S ribosome, bL35, bL36, hibernation, hibernation-promoting factor (YhbH), intact bL31, ribosome-associated inhibitor A (YfiA), ribosome-modulation factor

## Abstract

Kaltschmidt and Wittmann made significant advances in protein separation techniques by pioneering the two-dimensional polyacrylamide gel electrophoresis (2-D PAGE) method. However, it possessed two serious weaknesses. One was the extremely strong oxidizing power of the polyacrylamide gel, and the other was the excessively high pH of 4.5 of the second-dimensional gel. Consequently, it could not identify all the ribosomal proteins (r-proteins) of *Escherichia coli*. The radical-free and highly reducing (RFHR) 2-D PAGE overcame the first weakness by prerunning 2-aminoethanethiol HCl, possessing both the properties of a reducing agent and a radical scavenger. The second weakness was addressed by lowering the pH of the second dimension running buffer to 3.6. These improvements eliminated spot pattern noise and achieved the separation of low molecular weight proteins in the sample gel, establishing a new method of 2-D PAGE with high resolution and highly quantitative performance. Using RFHR 2-D PAGE, additionally, we discovered new ribosomal proteins bL35 and bL36, intact bL31, and identified all ribosomal proteins corresponding to the ribosomal genes of the *E. coli* genome. We also discovered the ribosomal hibernation state in bacteria and the essential hibernation factors. Analysis of total proteins in *E. coli* cells using the RFHR method revealed 65 spots exhibiting changes in protein content in the stationary phase.

## 1 Introduction

In 1953, Watson and Crick elucidated how the structure and function of DNA govern the phenomenon of heredity in living organisms. Subsequently, it became clear that mRNA conveys the genetic code from DNA to the ribosome. Consequently, elucidating the structure of the ribosome, the site of protein synthesis, became one of the major challenges of molecular biology in the 1960s. The fundamental physicochemical properties of *Escherichia coli* ribosomes, which consist of an rRNA + r-protein complex, have been established (Tissières et al., 1959).

### 1.1 Development of two-dimensional electrophoresis by Kaltschmidt and Wittmann

Identifying several dozen ribosomal proteins became a fierce competition among research groups. Disk electrophoresis was employed by Nomura and Traub (1968) (known as the Madison code; regarding the naming of ribosomal proteins) and by Traut et al. (1967) (known as the Geneva code), phosphocellulose chromatography by Craven et al. (1969) (known as the Uppsala code), carboxymethyl cellulose chromatography by Otaka et al. (1968), and 2-D PAGE (known as the Berlin code) by Kaltschmidt and Wittmann (1970a), Kaltschmidt and Wittmann (1970b). Kaltschmidt and Wittmann prevailed with the far higher resolution of their 2-D PAGE and, in 1970, 55 ribosomal protein spots were named, including bS1 to bS21 for the 30S subunit and uL1 to bL34 for the 50S subunit (Kaltschmidt and Wittmann, 1970b). Subsequently, L7 was identified as the acetylated bL12, L8 was identified as a complex of L7/bL12 and uL10, and L26 was found to be the same protein as bS20. Following removal of these duplicates, 52 ribosomal proteins were identified (Kaltschmidt and Wittmann, 1970b; Giri et al., 1984).

### 1.2 Discovery of new ribosomal proteins L35 and L36 by RFHR 2-D PAGE

As mentioned above, the two ribosomal proteins bL35 and bL36, which had been overlooked due to the limitations of the K–W method, were discovered in 1986 using RFHR 2-D PAGE, thereby completing the identification of all 54 ribosomal proteins in *E. coli* (Wada, 1986a; b; Wada and Sako, 1987).

### 1.3 The emergence and issues of O’Farrell’s isoelectric point 2-D PAGE

O'Farrell introduced the isoelectric focusing (IEF) method (O'Farrell, 1975) in 1975. This method aimed to establish a pH gradient from acidic to basic in a one-dimensional gel, in which proteins would halt migration upon reaching their isoelectric point (pI), concentrate, and form sharp spots. An IEF-based protein separation method was subsequently optimized and became widely used, facilitating proteome analysis studies. However, the pI range that can be resolved by the IEF is narrow (approximately pI 3–10); furthermore, a single protein could be identified from multiple spots, often exceeding ten (Berven et al., 2003). It was discovered that many quasi-stable stationary points, not merely isoelectric points, were generated.

### 1.4 High quantitative capability of RFHR 2-D PAGE

In contrast, RFHR 2-D PAGE, in which proteins migrate through gels maintained at constant pH in both the first and the second dimensions, allows each protein to converge into a single spot. Consequently, quantifying the amount of each protein is easily achieved by measuring the corresponding stained spot. Since the establishment of the method in 1986, improvements have been made to enable the separation and quantification of bacterial and cellular proteins. This method has excellent reproducibility, with each spot representing a single protein, and is highly effective for the quantitative analysis of both acidic and basic intracellular proteins. This article describes examples of an experiment for applying RFHR 2-D PAGE to *E. coli* total proteins.

## 2 Experimental methods

**FIGURE 1:**
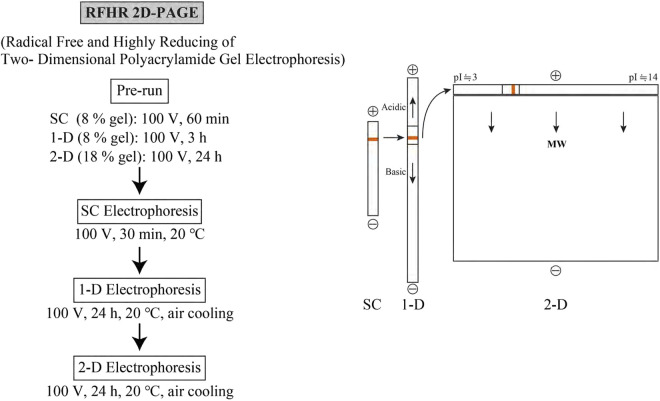
RFHR 2-D PAGE consisting of three polyacrylamide gels. The right side shows the order of operations for the sample charging gel (SC, preparing gel pieces containing concentrated proteins), the one-dimensional gel (1-D, primarily separation of proteins by electrical charge), and the two-dimensional gel (2-D, primarily separation of proteins by molecular size). The left side shows the electrophoretic conditions of each gel stage.

As shown in Figure 1, RFHR 2-D PAGE consists of three polyacrylamide gels. All gels are infused with 2-aminoethanethiol HCl, a radical scavenger and reducing agent, prior to protein electrophoresis (prerun). This removes free radicals and transforms the oxidizing gel environment into a strongly reducing one. Cysteine residues in proteins are maintained in a reduced state throughout electrophoresis by 2-aminoethanethiol HCl, and noise caused by cysteine dimerization is eliminated thoroughly. Therefore, one type of protein converges on a single spot. Prior to the first-dimensional electrophoresis, sample charging (SC) electrophoresis is performed to concentrate the protein sample using a leading ion and trailing ion. A sample gel fragment less than 1 cm in size containing the concentrated proteins is embedded into the one-dimensional gel. In the first-dimensional electrophoresis, proteins migrate mainly based on their charge magnitude: positively charged proteins move to the right and negatively charged proteins move to the left at pH 8.3. After the first-dimensional gel is laid atop the second-dimensional gel, the second-dimensional electrophoresis is performed at pH 3.6. Proteins migrate from top to bottom mainly based on their molecular size. Very small basic proteins also form normal spots. The completed two-dimensional gel is typically stained with Coomassie brilliant blue (CBB) G250 and destained. Protein spots on the two-dimensional gel are measured using a GS800 calibrated densitometer (Bio-Rad 232 Laboratories Inc., Hercules, CA, United States).

### 2.1 Sample preparations for RFHR 2-D PAGE

#### 2.1.1 Bacteria and growth

*E. coli* K12 W3110 wild and W3110 *ΔompT::Km* (Ueta et al., 2017) strains were grown in medium E (Vogel and Bonner, 1956) supplemented with 2% (w/v) hipolypeptone (Fujifilm Wako, Japan) and 0.5% (w/v) glucose (EP medium) (Wada, 1986a) at 37 °C and harvested at the log phase of growth or at the stationary phase. Collected cells were stored at – 80 °C.

#### 2.1.2 Fractionations (crude ribosome (CR), cell debris (CD), and post-ribosomal supernatant (PRS)) of disrupted cells

Crude ribosomes (CRs) were prepared from cellular extracts (CEs) according to the method of Noll et al. (1973) with slight modifications (Horie et al., 1981). Harvested cells were ground with equal volumes of quartz sand (FUJIFILM Wako), and the disrupted cells were extracted with buffer I [20 mM Tris-HCl (pH 7.6), 15 mM magnesium acetate, 100 mM ammonium acetate, and 6 mM 2-mercaptoethanol]. The homogenate was centrifuged at 9,000 × *g* for 15 min at 4 °C. The supernatant was saved, and the pellet was resuspended in buffer I. Subsequently, the suspension was centrifuged again under the same conditions, and the resulting pellet was collected as the cell debris (CD) fraction. The supernatants were combined as the CE fraction and were layered onto a 30% (w/v) sucrose cushion in buffer I and centrifuged in a 55.2 Ti rotor (Beckman, Fullerton, CA, United States) at 206,000 × *g* for 3 h at 4 °C. The pellet was resuspended in buffer I and used as the CR fraction, and the supernatant was used as the post-ribosomal supernatant (PRS) fraction.

#### 2.1.3 Ribosome preparations: high salt-washed ribosomes (HSRs)

HSRs were prepared as described by Horie et al. (1981). CRs were resuspended in buffer II [20 mM Tris-HCl (pH 7.6), 10 mM magnesium acetate, 1 M ammonium acetate, and 6 mM 2-mercaptoethanol]. After mixing for 1 h at 4 °C, the high-salt-washed suspension (20 mL) was layered onto a 30% (w/v) sucrose cushion in buffer II (10 mL) and centrifuged in a 55.2 Ti rotor (Beckman) at 206,000 × *g* for 4 h at 4 °C. The pellet was resuspended in buffer I and dialyzed against buffer I overnight. This suspension was used as HSR.

#### 2.1.4 Analysis of ribosomes by sucrose density gradient (SDG) centrifugation

Each ribosome sample was layered onto a 5%–20% (w/v) SDG in buffer I and centrifuged in an SW 40 Ti rotor (Beckman) at 25,000 × *g* for 20 h at 4 °C. The SDGs were prepared using a gradient maker (Gradient Mate 6T; BioComp Instruments, British Columbia, Canada). After centrifugation, the absorbance of each fraction was measured at 260 nm using a flow cell in a UV-1800 spectrometer (Shimadzu).

#### 2.1.5 Preparation of proteins from the CR, PRS, and CD fractions

Proteins were prepared from the CR, PRS, and CD fractions by the acetic acid method (Hardy et al., 1969). After dialysis against 2% (v/v) acetic acid three times, they were lyophilized by Labconco (Kansas City, MO, United States) and stored at −80 °C.

### 2.2 Solutions for RFHR 2-D PAGE

#### 2.2.1 The stocked concentrated buffers

Gel solutions and electrode buffers were prepared each time, using stocked concentrated buffers as follows. Sample charging (SC) buffer (50×): 24 mL 5 N KOH, 74 mL glacial acetic acid, and water in 200 mL; 1-D buffer (4×): 32.0 g EDTA-2Na (Dojindo Kumamoto Japan, Guaranteed Reagent (GR)), 128 g boric acid, 194.5 g Tris, and water in 1 L; 2-D gel buffer (10×): 96 mL 5 N KOH, 523 mL glacial acetic acid and water in 1 L; 2-D electrode buffer (10×): 16.0 mL glacial acetic acid, 150 g glycine (FUJIFILM Wako GR), and water in 1 L.

#### 2.2.2 The solution for SC

The gel solution consists of 42.0 g urea (FUJIFILM Wako GR), 7.0 g acrylamide (FUJIFILM Wako GR), 0.125 g bisacrylamide (FUJIFILM Wako GR), 0.3 mL N, N, N′, N′-tetramethylethylenediamine (TEMED) (FUJIFILM Wako GR), 2 mL SC buffer (50×), and water in 100 mL. For gelation, 1/100 volume of 10% (w/v) ammonium peroxodisulfate (ammonium persulfate) (FUJIFILM Wako GR) was added. The anode prerun buffer consists of 45.0 g urea, 1.0 g 2-aminoethanethiol HCl [TCI GR, Tokyo, Japan (also known as 2-mercaptoethylamine HCl)], 2 mL SC buffer (50×), and water in 100 mL. The cathode prerun buffer is the same as the anode prerun buffer, except there is no addition of 2-aminoethanethiol HCl. The anode run buffer consists of 24.0 g urea, 3.5 g cysteine-HCl (FUJIFILM Wako GR), 10 mL 2-D electrode buffer (10×), and water in 100 mL. The cathode run buffer consists of 36.0 g urea, 10 mL 2-D gel buffer (10×), and water in 100 mL.

#### 2.2.3 Solutions for 1-D

The gel solution consists of 42.0 g urea, 7.0 g acrylamide, 0.125 g bisacrylamide, 0.3 mL TEMED, 25 mL 1-D buffer (4×), and water in 100 mL. 1-D gels were gelated in the same manner as the SC gels. The anode prerun and run buffers consist of 45.0 g urea, 1.0 g 2-aminoethanethiol HCl, 25 mL 1-D buffer (4×), and water in 100 mL. The cathode prerun and run buffers are the same as the anode prerun buffer, except that 2-aminoethanethiol was not added.

#### 2.2.4 Solutions for 2-D

The gel solution consists of 420 g urea, 170 g acrylamide, 4.7 g bisacrylamide, 5.8 mL TEMED, 100 mL 2-D gel buffer (10×), and water in 1 L. For gelation, 1/30 volume of 10% ammonium peroxodisulfate was added. The anode prerun buffer consists of 168 g urea, 2.0 g 2-aminoethanethiol HCl, 40 mL 2-D gel buffer (10×), and water in 400 mL. The cathode prerun buffer consists of 40 mL 2-D gel buffer (10×) and water in 400 mL. The anode run buffer consists of 168 g urea, 14 g cysteine-HCl, 40 mL 2-D electrode buffer (10×), and water in 400 mL. The cathode run buffer consists of 1.0 mL hydrochloric acid [HCl, 35.0–37.0% (Titration), FUJIFILM Wako], 40 mL 2-D electrode buffer (10×), and water in 400 mL. HCl was added to promote the release of r-proteins from the interfaces between the tailing and leading ions.

### 2.3 Procedures for electrophoresis

#### 2.3.1 Preparations for sample loading

A lyophilized mixture of ribosomal proteins was dissolved in 40-fold-diluted 1-D buffer (4×) containing 8 M urea and 0.2 M 2-mercaptoethanol. After incubation at 40 °C for 30 min, 1/25 volume of SC buffer (50×) was added. Pyronine-G (Merck KGaA, Germany) and, if necessary, recrystallized acridine orange (FUJIFILM Wako) were added to the sample solution as a migration marker for SC. The optimal amount of r-proteins applied to a gel was 0.3–1.0 mg (20 mg/mL). CD and PRS solutions were also treated the same as the r-protein solution.

#### 2.3.2 SC electrophoresis

**FIGURE 2:**
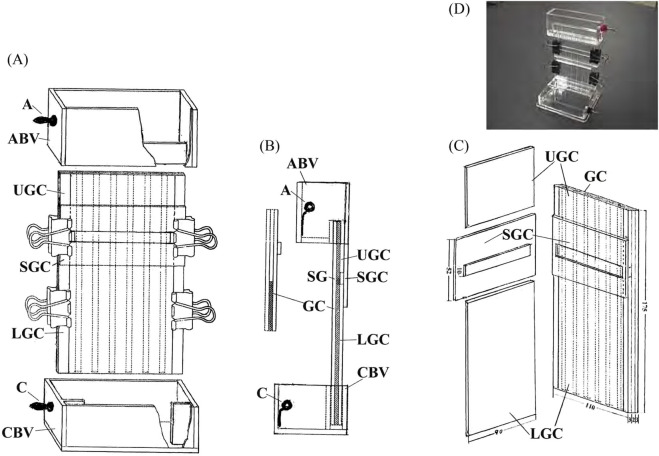
One-dimensional and sample charging apparatus. **(A)** A perspective view of the 1-D apparatus. The upper part: anode (A) and anode buffer vessel (ABV). The middle part: gel container (GC), sample gel cover (SGC), upper gel cover (UGC), lower gel cover (LGC), and four double clips. The lower part: cathode **(C)** and cathode buffer vessel (CBV). **(B)** The side view of the 1-D and the sample charging apparatus. The left short gel container is used for sample charging. **(C)** Perspective view of the 1-D gel container, sample gel cover, upper gel cover, and lower gel cover. A photograph of the current 1-D apparatus is attached. **(D)** Photograph of the 1-D apparatus.

SC electrophoresis was introduced to prepare sample gel pieces for 1-D electrophoresis. The apparatus consisted of three parts: an anode (A) and a cathode (C) buffer vessel (anode buffer vessel (ABV) and cathode buffer vessel (CBV)) that were the same as those of the 1-D electrophoresis (Figure 2) and a short gel container (GC). The gel container had six gel chambers, each of which had a 60 mm height and a 2 × 5 mm^2^ cross sectional area. It was bonded to the anode buffer vessel. The SC-gel solution was poured into the gel chambers up to a height of 40 mm, overlaid with water, and gelated. Prerun was performed at 100 V for 1 h using the SC-prerun buffers. At the end of the prerun, sample solutions were added to the SC gels and overlaid gently with the anode run buffer. SC electrophoresis was performed at 100 V for approximately 10–30 min. Pyronine-G and acridine orange in the sample solutions were concentrated rapidly and migrated into the gels. Proteins followed them. When pyronine-G and acridine orange had run inside the gels for approximately 10 mm, the run was finished. The gels were carefully loosened and taken out of the gel chambers by injecting SC anode run buffer through an injecting needle into the interfaces between the gels and the gel chambers. Sample gel pieces containing proteins were obtained by cutting the gels with a razor at a position immediately below the band of pyronine-G and within a length of 10 mm. The sample gel pieces were used for 1-D electrophoresis.

#### 2.3.3 1-D electrophoresis

As shown in Figure 2, the apparatus also consisted of three parts [(A), (B), and (C)]. The open gel container (GC) had six gel chambers, each of which had a 175 mm height and a 2 × 5 mm^2^ cross sectional area. The open gel container is covered by the sample gel cover, the upper gel cover, and the lower gel cover. It was fixed with four double clips, and the interface between the gel container and the window cover was sealed using 1-D gel solution, as described above. The interface between the gel container and the anode buffer vessel (ABV) was also sealed in a similar manner (Figures 2A,B). After the bottoms of gel chambers were gelated with 1-D gel solution, 1-D gel solution was poured to fill the length of the gel chambers and gelated. A prerun was performed at 100 V for 3 h. Thereafter, the sample gel cover was opened, and the exposed parts of the gels were cut out with a ground spatula to the same length as the sample gel pieces. The sample gel pieces were inserted into the spaces, and a small amount of 1-D anode run buffer was added onto them to remove air bubbles. The sample gel cover was closed again without gelation. 1-D electrophoresis was performed at 100 V for approximately 24 h at room temperature with air cooling. At the end of the electrophoresis, the 1-D gel and the sample gel pieces were taken out with a spatula. The excess parts of the 1-D gels were cut to the same length as the width of the 2-D gels. The ratio of the length of the cathode side gel to the length of the anode side gel was 2.

#### 2.3.4 2-D electrophoresis

**FIGURE 3:**
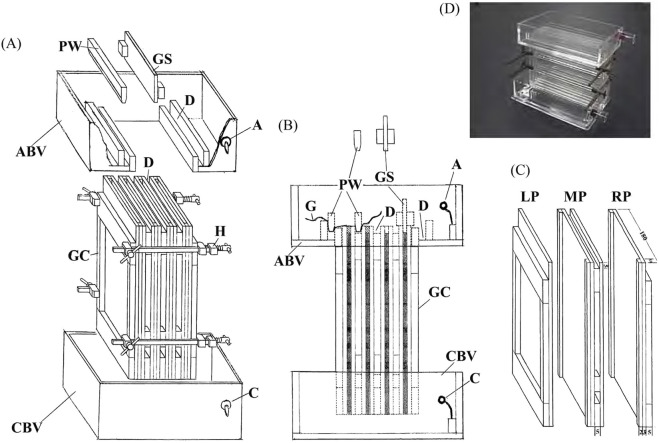
Two-dimensional apparatus. **(A)** Perspective view. The upper part: anode (A), anode buffer vessel (ABV), four 1-D gel spacers (GS), five pressing wedges (PW), and two grooves (D). The middle part: the gel container (GC) consisting of three middle parts, two side parts, and four hataganes (Japanese bar clamps) (H). The anode buffer vessel and the gel container have three grooves (D) for inserting pressing wedges. The lower part: cathode (C) and cathode buffer vessel (CBV). **(B)** The side view. A piece of fine cotton gauze (G) or fabric for women’s nylon stockings is stretched strongly and pressed into the grooves with the pressing wedges. The hataganes are not shown. The 1-D and 2-D gels are indicated by light and dense hatching, respectively. **(C)** Perspective view of the gel container: its left part (LP), middle parts (MP), and right part (RP). Sizes are indicated in millimeters. A photograph of the current 2-D apparatus is attached. **(D)** Photograph of the 2-D apparatus.

The apparatus for 2-D electrophoresis consisted of two buffer vessels (ABV and CBV) and a gel container (GC) (Figure 3). The gel container was an assembly of a left part (LP), three middle parts (MPs), and a right part (RP) (Figure 3C). When assembled, the five parts form four gel chambers with 140 mm height, 160 mm width, and 2 mm thickness. As shown in Figure 3A, all the parts of the gel container were firmly assembled using four hataganes (H) (Japanese bar clamps). Thereafter, the gel container was inserted into the anode buffer vessel (ABV) and set on the 2-D gel dish. A 30-mL aliquot of the 2-D gel solution was added to the 2-D gel dish and gelated to make the bottoms of the 2-D gels. The interfaces between the 2-D gel anode buffer vessel and 2-D gel container were sealed in the same way. Gel chambers and side grooves were filled with 2-D gel solution. “1-D gel spacers (GS)” (Figure 3) were inserted into the tops of the gel chambers to leave 5 mm depth spaces for setting the 1-D and sample gels (Figure 3B). After the gelation, the spacers and excess gels were removed, and the prerun was performed at 100 V for 24 h with air cooling. Thereafter, the 1-D and sample gels were put on the 2-D gels, carefully avoiding bubbles in their interfaces. A piece of fine cotton gauze (G), or fabric from a woman’s nylon stockings, was wetted with anode run buffer and used to cover the 1-D and sample gels. The gauze was pressed by five pressing wedges (PW) that were inserted into the five grooves (D) (Figure 3B). The gauze was thereby stretched strongly and pressed the 1-D and sample gels against the 2-D gels. In this way, good contact between the 1-D and 2-D gels could be made without using gelation. The 2-D electrophoresis was performed at 100 V for approximately 24 h at room temperature with air cooling. Pyronine-G remaining in the 1-D gel was used as a 2-D front marker. When it reached the bottom, the 2-D run was finished.

#### 2.3.5 Staining and destaining of gel

The 2-D gels were carefully removed from the gel chamber and stained with 0.25% (w/v) Coomassie Brilliant Blue G 250 (FUJIFILM Wako) in 9% (v/v) acetic acid for 30 min. They were then destained by shaking in the solution containing 3% (v/v) acetic acid and 30% (v/v) ethanol, followed by repeated destaining in 2% (v/v) acetic acid.

### 2.4 Analysis of electrophoresis gel spots

#### 2.4.1 Determination of the copy number of ribosomal proteins and ribosome-binding factors

The stained 2-D gel patterns were scanned using a GS800 calibrated densitometer (Bio-Rad Laboratories Inc., Hercules, CA, United States). The optical density (OD)/molecular weight (MW) of each r-protein was calculated. Among them, the values of the ten proteins (S4, S5, S7, S13, L18, L22, L24, L25, L27, and L29) were designated as unit copy proteins (Hardy, 1975; Tal et al., 1990). The average of these OD/MW values was set to 1, and the copy number of each protein was expressed as a ratio relative to the unit copy proteins. The OD/MW values of each r-protein and ribosome-binding factor were converted to copy numbers.

#### 2.4.2 Gene identification of proteins

A spot was punched out from the two-dimensional gel and placed into a microtube for outsourcing to a contractor for protein identification via mass spectrometry. Sufficient protein content within a single spot enables protein identification.

## 3 Experimental results

### 3.1 Proteins of CD and PRS fractions in *Escherichia coli*

**FIGURE 4:**
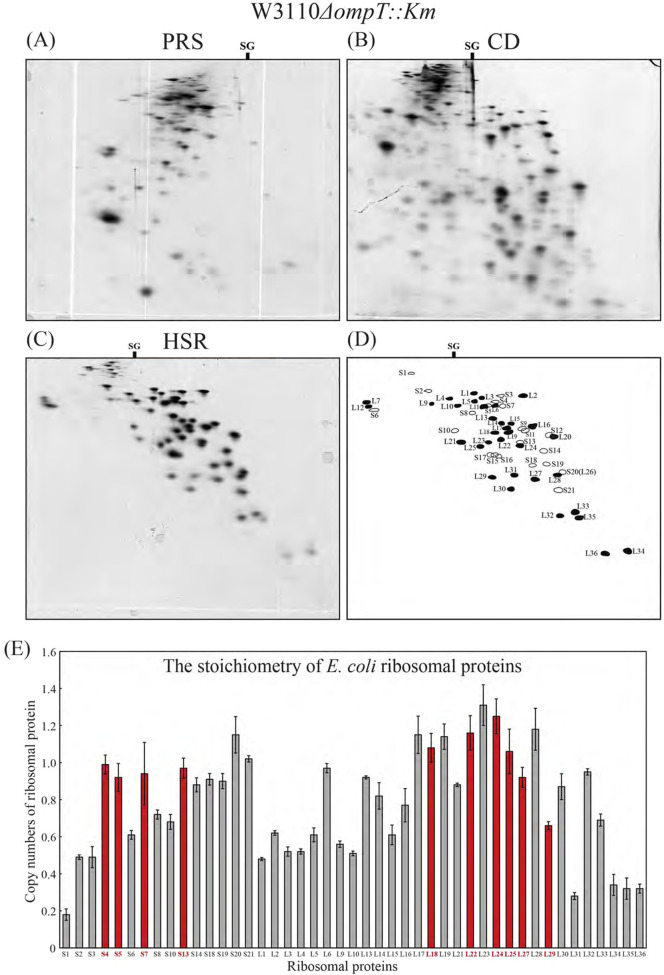
Analysis by the RFHR method of total proteins and ribosomal proteins in *Escherichia coli*. Three fractions (CR, PRS, and CD) from *E. coli* W3110 *ΔompT::Km* were prepared as described in Section 2.1.2. The proteins contained in each fraction were analyzed by RFHR 2-D PAGE. **(A)** Proteins of the PRS fraction. **(B)** Proteins of the CD fraction. **(C)** CR-protein fraction. **(D)** Scheme for **(C)**. SG shows the position of the sample gel. Black spots correspond to 50S subunit proteins, and white ones to 30S subunit proteins. **(E)** Reproducibility and quantitative accuracy of the RFHR method: The stoichiometry of ribosomal proteins in *E. coli* W3110 wild strain was determined by CBB G250 staining of RFHR 2-D PAGE performed on three gels, using the CR fraction prepared from log-phase cultured cells of *E. coli* W3110 wild strain (see Section 2.1). The vertical axis shows the average copy number of 45 r-proteins with error bars. The ten r-proteins used as unit copies are indicated in red. The horizontal axis represents the r-proteins.

As described in Sections 2.1.2, 2.1.5, proteins of the CD and PRS fractions were prepared and separated by RFHR 2-D PAGE. Most proteins within cells belong to the PRS fraction. Basic proteins are extremely rare in this category, with most being acidic rather than weakly basic (Figure 4A). As shown in Figure 4B, proteins belonging to the CD fraction are widely distributed across both acidic and basic regions. The basic region contains proteins derived from the nucleoid, along with ribosomal proteins that have detached from ribosomes, which are present in varying numbers.

### 3.2 Identification of new ribosomal proteins bL35 and bL36 in *Escherichia coli*

As described in Sections 2.1.2, 2.1.3, the CR fraction of *E*. *coli* W3110 *ΔompT::Km* cells was treated with a high salt concentration, the HSRs were prepared, and the r-proteins of HSR were separated by RFHR 2-D PAGE (Figure 4C). In addition to the 52 species identified by the K–W method, bL35 and bL36 were newly discovered by RFHR 2-D PAGE, bringing the total number of *E. coli* ribosomal proteins to 54, which corresponds to the ribosomal protein genes in the *E*. *coli* genome (Wada, 1986a; b; Wada and Sako, 1987). bL35 possesses one cysteine residue. In the conventional K–W method, this cysteine is highly susceptible to oxidation, leading to the formation of disulfide bridges to other proteins during electrophoresis, rendering the original spot undetectable. However, the RFHR method completely prevented oxidation, resulting in the formation of distinct spots. Oxidized gel (K–W method) and reduced gel (RFHR method) are compared in Supplementary Figure S1. In the oxidized gel (Supplementary Figure S1B), not only was L35 undetectable, but spots for all cysteine-containing proteins were reduced. Numerous noise spots appeared due to cysteine dimerization. In contrast, the reduced gel (Supplementary Figure S1A, RFHR method) showed the intrinsic spots for all ribosomal proteins, including L35 at its single-copy size. No noise was observed. At pH 4.5 of the second-dimensional electrophoresis buffer in the K–W method, the migration rate of bL36 exceeded that of the trailing ion, preventing the formation of a normal spot, as shown in Supplementary Figure S1B. However, at pH 3.6 of the 2-D buffer in RFHR 2-D PAGE, it migrated more slowly than the trailing ion and formed its characteristic spot, as shown in Supplementary Figure S1A. *E. coli* is the first organism for which all ribosomal proteins have been identified. The nomenclature and location on the RFHR 2-D PAGE are shown in Figure 4D. Proteins of the 50S subunit are designated with an L prefix (black circle), while proteins of the 30S subunit are designated with an S prefix (white circle).

### 3.3 Discovery of the intact bL31 protein in *Escherichia coli*

**FIGURE 5:**
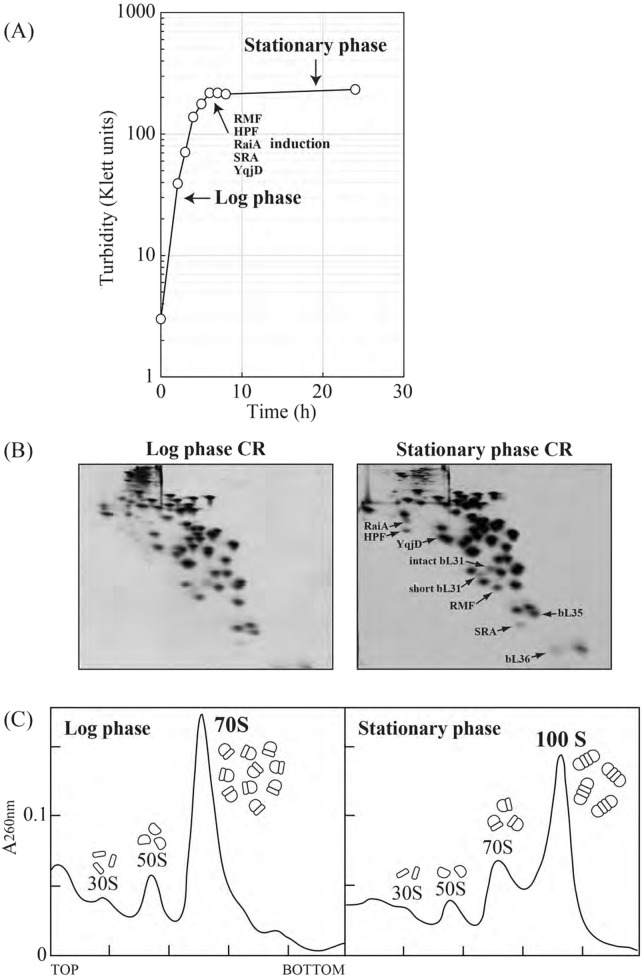
Appearance of hibernation factors and 100S ribosomes during the stationary phase in *E*. *coli*. **(A)** Growth curve of *Escherichia coli* W3110 wild-type strain in EP medium at 37 °C. Cell growth was monitored via turbidity (Klett units). The vertical axes show normal logarithmic values. The ribosome-binding proteins ribosome-modulation factor (RMF), hibernation-promoting factor (HPF), ribosome-associated inhibitor A (RaiA), stationary phase-induced ribosome-associated protein (SRA), and YqjD are expressed immediately after the end of the log phase. **(B)** Protein spot patterns of crude ribosomes of *Escherichia coli* cells in log and stationary phases using RFHR 2-D PAGE. RMF, HPF, RaiA, SRA, YqjD, short L31, intact L31, bL35, and bL36 spots are indicated by arrows. **(C)** The ribosome profiles of *Escherichia coli* in the log and stationary phases were obtained through sucrose density gradient centrifugation. The 100S peak appears in the stationary phase. Conversely, the 70S peak decreases.

The W3110 *ΔompT::Km* strain used here is a mutant lacking outer membrane protease 7. We discovered that in the wild-type strain, when cells are lysed to prepare the ribosomal proteins, they encounter active protease 7, which cleaves the C-terminal 7 amino acids of bL31 (Ueta et al., 2017; Ueta et al., 2005; Wada et al., 2023). Most previously reported findings on ribosomal function are based on results from ribosomes with a deficient short bL31 and are therefore unreliable. By employing the *ΔompT::Km* mutant strain, these weaknesses were resolved. Figure 4C shows that the deficient short bL31 does not exist; only the intact L31 is found. (In the right panel of Figure 5B, which shows W3110 wild-type cells, intact and deficient short bL31 spots are visible.)

### 3.4 Reproducibility and quantitative accuracy of RFHR 2-D PAGE

To verify the reproducibility and quantitative accuracy of RFHR 2-D PAGE, the copy number of r-proteins in *E. coli* W3110 wild-type cells was determined. A 0.5-mg sample of crude ribosome (CR) was applied to each of three gels, and the standard RFHR method was performed. Of the 54 r-proteins, nine spots overlapped (extending the electrophoresis time avoids this overlap), so only 45 were analyzed under the conditions. The copy number was calculated as described in Experimental Methods (Section 2.4.1). Figure 4E shows the average copy number of each ribosomal protein across the three experiments and its standard deviation. The ten proteins (S4, S5, S7, S13, L18, L22, L24, L25, L27, and L29) designated as unit copy proteins are illustrated by red bars. Although the OD measured by CBB (G250) staining is significantly influenced by the content of basic amino acids, which are abundant in ribosomal proteins, the copy number of these ribosomal proteins (red bars) is relatively close to one. The coefficient of variation (CV) for the 45 proteins is >10% for 7/45 (16%), 5–10% for 19/45 (42%), and <5% for 19/45 (42%). This value demonstrates the high quantitative capability of the RFHR method for proteins. Compared to O'Farrell’s isoelectric point 2-D PAGE, in which multiple spots for ribosomal proteins such as RpsA (S1), RpsB (S2), and RplI (L9) appear, the RFHR method produces a single spot for each of these proteins (Supplementary Figure S2). No reports indicate that these ribosomal proteins are modified. Furthermore, RpsF (S6) is known to undergo modification where glutamic acid is added at the C-terminus, and the multiple spots of this protein can be confirmed on the gels of both electrophoresis methods. These facts indicate that the results obtained by the RFHR method are more accurate.

### 3.5 Discovery of bacterial hibernation and hibernation proteins

Five proteins (RMF, HPF, RaiA (YfiA), YqjD, and SRA) in *E. coli* specifically expressed during the stationary phase and binding to ribosomes were identified using RFHR 2-D PAGE (Wada et al., 1990; Agafonov et al., 1999; Maki et al., 2000; Izutsu et al., 2001; Yoshida et al., 2012) (Figures 5A,B). As shown in Figure 5C, during the stationary phase, the ribosome-modulation factor (RMF) (Wada et al., 1990) and hibernation-promoting factor (HPF) bind to the ribosome, forming the 70S ribosomal dimer, the 100S ribosome (Wada, 1998; Yoshida and Wada, 2014). First, the 70S complex bound to RMF forms a dimer, becoming the 90S ribosome. Subsequently, upon HPF binding, the 90S matures into a 100S ribosome (Ueta et al., 2005). RaiA (YfiA) is a homolog of HPF (YhbH). RaiA has the same N-terminal domain topology as HPF, and it has a flexible C-terminal tail (Rak et al., 2002; Polikanov et al., 2012). Analysis of deletion mutants of HPF and/or RaiA revealed that RaiA inhibits 100S ribosome assembly mediated by RMF (Ueta et al., 2005). The 30S subunit component bS1 is involved in 100S maturation (Beckert et al., 2018). RMF not only inhibits mRNA recruitment by binding to the 30S platform, but it also supports the fixation of the bS1 conformation. Furthermore, RMF is known to compete with the initial target region of RNase R, which is involved in the ribosomal RNA degradation (Dimitrova-Paternoga et al., 2024). HPF binds to the mRNA channel of the 30S subunit. Together with RMF, it inhibits mRNA recruitment and stabilizes the 30S head and body domains (Polikanov et al., 2012). On the other hand, the N-terminal domain of RaiA interacts with the same binding region of the 30S subunit as HPF (Polikanov et al., 2012), and its flexible C-tail overlaps with the RMF-binding region. Thus, RaiA competes with both HPF and RMF, and the RaiA-bound hibernating ribosome cannot dimerize (Rak et al., 2002; Polikanov et al., 2012). In addition, YqjD is involved in ribosomal membrane association (Yoshida et al., 2012). Stationary-phase-induced ribosome-associated (SRA) binds specifically to ribosomes during the stationary phase, but its structure and function remain unclear (Izutsu et al., 2001). 100S suppresses protein synthesis yet paradoxically extends the lifespan of *E. coli*. That is, it confers a hibernation function upon *E. coli*. Recently, it has become clear that this type of hibernation via 100S formation is widespread among bacteria (Ueta et al., 2013; Ueta et al., 2024; Tanzawa et al., 2026). In these studies of various bacteria, the new hibernation proteins have been discovered by the RFHR method.

### 3.6 Changes in protein content in the stationary phase state

**FIGURE 6:**
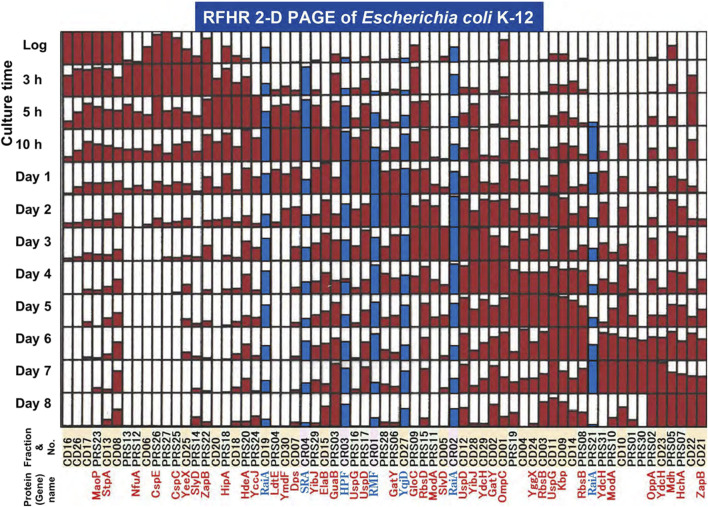
Changes in protein levels during the stationary phase of 65 protein spots. In this experiment, each gel was loaded with the same total amount of protein, so the spot intensity directly reflects the proportion of that protein within the total proteome. For each spot, the densitometric intensity was scaled to its own maximum value across all sampling points; thus, a maximum bar height corresponds to the highest observed signal for that spot, making it possible to observe how the intensity of each spot changes over the course of the experiment. The blue bar shows ribosome-binding proteins, and the other red bar shows proteins free from ribosomes. Spot numbers and protein names are shown on the horizontal axis. Culture times are shown on the perpendicular axis. This figure was modified from Figure 4 of the article published by Yoshida et al. (2019).

The total protein from *E. coli* K12 cells was fractionated into three fractions: CR, PRS, and CD. Each fraction was analyzed by RFHR 2-D PAGE. Approximately 600 spots were detected; of these, 65 showed a clear time-dependent change in intensity during the stationary phase (Figure 6). Because each gel was loaded with the same amount of protein, the red and blue bars in Figure 6 represent the relative intensity of an individual spot normalized to its maximal value (set to 1) across all sampling points. According to the growth curve shown in Figure 5A, we defined the period from inoculation up to 5 h as the growth phase and all later times (10 h ∼ Day 8) as the stationary phase. Twenty of the variable spots reach their maximum during the growth phase, whereas the remaining 45 achieve their peak in the stationary phase. Their appearance is heterogeneous: some of the stationary-phase proteins emerge at a specific time point and disappear soon after, showing a relatively narrow window of expression, while the others are distributed over a broader portion of the stationary phase, even though their relative abundance shifts among the three subcellular fractions (Yoshida et al., 2019). Nine spots were detected for the first time only after entry into the stationary phase, showing stage-specific transcriptional regulation and/or physiological remodeling as the culture ages. Among the 65 dynamic spots, five correspond to ribosome-binding proteins as described above, while the rest were free from ribosomes (Figure 6). The combination of 65 proteins in the stationary phase undergoes constant change. How they relate to hibernation is an interesting question.

### 3.7 Preservation of RFHR 2-D PAGE

**FIGURE 7:**
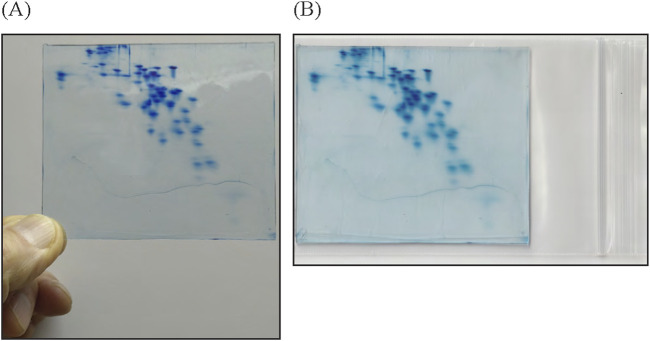
Preserved plastic plate prepared from the original gel **(A)** and kept in a soft bag for preservation **(B)**. Following the procedure in Section 3.6, the plastic plate was made by leaving the original gel at room temperature for several days. The size of the plastic plate is approximately 90 mm × 77 mm, reduced from the 160 mm × 135 mm size of the original gel. The plasticized gel in **(A)** has hardened, so it can be held by its edge.

We have successfully developed a plastic formulation for long-term storage of RFHR 2-D PAGE gel. Destained RFHR 2-D PAGE gel used for analysis is placed flat in 2% (v/v) acetic acid, on a plastic mesh at room temperature. After several days, the gel shrinks and solidifies in a distorted state. The solidified gel is placed in a plastic bag, sealed to ensure dryness, and immersed in water at 80 °C for 1 min. The gel softens and must be immediately removed from the soft plastic bag. Then, the gel is sandwiched between flat plates, and pressure is applied. Figure 7A shows a real plastic plate that is approximately half the length and one-quarter of the area of the original gel. Figure 7B shows the plastic plate in a soft bag for preservation. The plastic plate and its spot pattern can be preserved semi-permanently. When the shrunken plastic plate is immersed in 2% (v/v) acetic acid for 1 day, it reversibly returns to the size of the original gel.

## 4 Discussion

RFHR 2-D PAGE inherits the excellent spot separation capability of the K–W method. Furthermore, by prerunning 2-aminoethanethiol HCl, which acts as both a reducing reagent and a radical scavenger on all gels prior to migration of proteins, RFHR 2-D PAGE prevented cysteine oxidation (dimerization) and eliminated noise generation, as shown in Supplementary Figure S1. Optimizing the buffer conditions proved to be another key factor in the overall performance of RFHR 2-D PAGE. By employing techniques such as sample charging electrophoresis and by performing the second-dimensional electrophoresis at a low pH of 3.6, RFHR 2-D PAGE is shown to be an excellent method that enables the analysis of all proteins soluble in buffers containing 8 M urea (Figures 4A–C). These refinements directly enabled the detection of ribosomal proteins that have been missed by the conventional K–W method, such as bL35 and bL36 (Kaltschmidt and Wittmann, 1970b; Giri et al., 1984).

O'Farrell’s isoelectric point 2-D PAGE is relatively simple and widely used because commercially available, ready-made gels and equipment are readily accessible (Thermo Fisher Scientific Inc., United States). However, it has the drawback of unmodified protein spots splitting and appearing and is fundamentally only applicable to proteins with isoelectric points (pI) ranging from 3 to 10 (Berven et al., 2003). The 2-dimensional fluorescence difference gel electrophoresis (2-D-DIGE), although excellent for quantitative comparison of expression levels (Meleady, 2018), still relies on an IEF first dimension, so the problems of spot splitting and restricted pI range cannot be avoided. In contrast, the RFHR method has neither the spot-splitting artifact nor the pI limitation, allowing detection of both highly basic and acidic proteins without the constraints inherent to IEF-based approaches (Supplementary Figure S2).

The detection sensitivity of proteins using these electrophoresis methods fundamentally depends on the staining reagents (CBB G250: 50–100 ng/spot, silver staining: 0.1–1 ng/spot, fluorescent staining: 0.5–2 ng/spot). Mass spectrometry is primarily used for protein identification and is highly sensitive, capable of detecting proteins at the pg to fg levels (Momenbeitollahi et al., 2021). However, mass spectrometry is relatively challenging for protein quantification; therefore, coupling electrophoretic separation with LC-MS/MS is essential for effective proteome analysis.

A practical advantage of RFHR 2-D PAGE is that it requires only inexpensive reagents; no pre-cast gels or costly fluorescent dyes are needed, making the whole workflow low-cost compared with commercial IEF and DIGE kits. Furthermore, by combining these approaches with powerful gene expression analysis tools such as quantitative PCR (qPCR) and RNA-seq, it becomes possible to identify post-transcriptional regulation and post-translational modifications (Yoshida et al., 2019).

Because RFHR 2-D PAGE can quantitatively analyze proteins across an extremely wide pI range and it provides a comprehensive view of cellular proteins, encompassing not only highly basic ribosomal components but also strongly acidic proteins. As ribosome analysis expands to diverse bacterial species, RFHR 2-D PAGE has already been used to characterize ribosomal proteins and their hibernation factors (Ueta et al., 2024). This method is expected to become valuable for studying species- or tissue-specific ribosomal and ribosome-associated proteins in many additional bacteria and in eukaryotic organisms.

## Data Availability

The original contributions presented in the study are included in the article/Supplementary Material, further inquiries can be directed to the corresponding authors.
