# Supplementary material for: Analysis of proteins by a radical-free and highly reducing method of two-dimensional polyacrylamide gel electrophoresis
Source: Front Mol Biosci. 2026 Apr 24;13:1777271. doi: 10.3389/fmolb.2026.1777271 (PMC13152769; doi:10.3389/fmolb.2026.1777271)
Supplement: Supplementary file 1 [file Supplementaryfile1.docx]

Supplementary Material


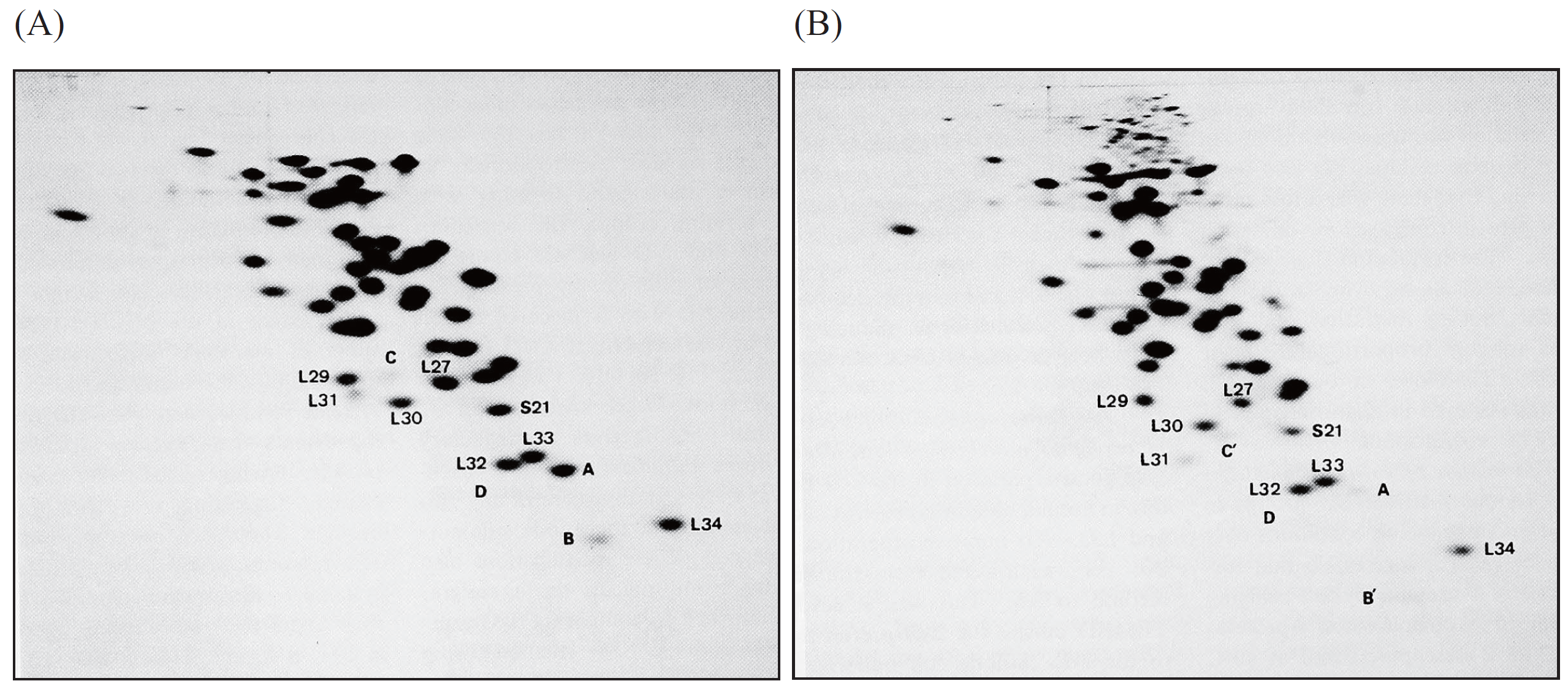


**Supplementary Figure 1.** Spot patterns of r-proteins prepared from high salt washed 70S ribosomes of E. coli strain Q13. (A) Under the reducing conditions (RFHR method). Electrophoresis was performed as described in "Experimental Methods." 0.6 mg/gel of r-protein was used. (B) Under the non-reducing conditions (K-W method). The electrophoresis was performed in the same way as (A) except using no thiol reagents and no incubation prior to sample charging. The spots of the proteins (A (bL35), B and B’(bL36), C and C’ (intact L31), D (SRA)) and the vicinal ones of r-proteins (bL27, uL29, uL30, bL31 (short L31), bL32, bL33, bL34 and bS21) are shown (Wada, 1986a).

**Supplementary Figure 2.** Comparison of RFHR 2-D PAGE and O'Farrell's Isoelectric Point 2-D PAGE. (A) Gel image showing *E. coli* proteins analyzed by the RFHR method (CBB staining). (B) Gel image showing *E. coli* proteins analyzed by the O'Farrell method (Silver staining).
